# Bystander preference for naloxone products: a field experiment

**DOI:** 10.1186/s12954-023-00904-9

**Published:** 2023-11-28

**Authors:** Katherine R. Marks, Douglas R. Oyler, Justin C. Strickland, Jody Jaggers, Monica F. Roberts, Dustin K. Miracle, Chase Barnes, Feitong Lei, Amanda Smith, Eric Mackin, Martika C. Martin, Patricia R. Freeman

**Affiliations:** 1 Department of Behavioral Science, College of Medicine, University of Kentucky, Lexington, KY USA; 2 Department for Behavioral Health, Developmental and Intellectual Disabilities, Cabinet for Health and Family Services, 275 E. Main Street, Frankfort, KY 40621 USA; 3 Department of Pharmacy Practice and Science, College of Pharmacy, University of Kentucky, Lexington, KY USA; 4 Department of Psychiatry and Behavioral Sciences, Johns Hopkins University School of Medicine, Baltimore, MD USA; 5 Kentucky Pharmacy Education and Research Foundation, Frankfort, KY USA; 6 Substance Use Priority Research Area, University of Kentucky, Lexington, KY USA; 7 Kentucky Department for Public, Health Division of Public Health Protection & Safety, Frankfort, KY USA; 8 Department of Biostatistics, College of Public Health, University of Kentucky, Lexington, KY USA

**Keywords:** Overdose education, Naloxone distribution, Opioid reversal agents, Bystander overdose response

## Abstract

**Background:**

Bystander administration of naloxone is a critical strategy to mitigate opioid overdose mortality. To ensure bystanders’ willingness to carry and administer naloxone in response to a suspected overdose, it is critical to select products for community distribution with the highest likelihood of being utilized. This study examines bystanders’ preference for and willingness to administer three naloxone products approved by the FDA for bystander use and identify product features driving preference.

**Methods:**

The population was a convenience sample of individuals who attended the Kentucky State Fair, August 18–28, 2022, in Louisville, Kentucky. Participants (*n* = 503) watched a standardized overdose education and naloxone training video, rated their willingness to administer each of three products (i.e., higher-dose nasal spray, lower-dose nasal spray, intramuscular injection), selected a product to take home, and rated factors affecting choice.

**Results:**

After training, 44.4% chose the higher-dose nasal spray, 30.1% chose the intramuscular injection, and 25.5% chose the lower-dose nasal spray. Factors most influencing choice on a 10-point Likert scale were ease of use (9 [7–10]), naloxone dose (8 [5–10]), and product familiarity (5 [5–9]).

**Conclusions:**

Bystanders expressed high willingness to administer all studied formulations of naloxone products. Product choice preference varied as a function of product features. As the number and variety of available products continue to increase, continuous evaluation of formulation acceptability, in addition to including individuals with lived experience who are receiving and administering overdose reversal agents, is critical to support adoption and save lives.

**Supplementary Information:**

The online version contains supplementary material available at 10.1186/s12954-023-00904-9.

## Background

Bystander administration of naloxone is a critical strategy to mitigate opioid overdose mortality, which is recognized as a public health emergency in the USA [1]. Naloxone, an opioid antagonist, can decrease the risk of a fatal overdose by reversing and blocking the effects of opioids if administered in time [2]. The Surgeon General of the United States Public Health Service has recommended that, in addition to individuals at risk of overdose, family, friends, and community members who encounter people at risk of overdose should know how to use naloxone and keep it within reach [3]. Community overdose education and naloxone distribution (OEND) programs increase access to naloxone and reduce harm from opioid use, and communities implementing OEND programs have lower rates of opioid overdose mortality than those without established programs [4, 5].

Various naloxone products are now approved by the United States Food and Drug Administration (FDA). Off-label naloxone in prefilled 2-mg/ml syringes adapted for nasal administration comprised most of the dispensed naloxone prescriptions [6] prior to the approval of the Evzio^®^ autoinjector in 2014 and Narcan^®^ 4-mg nasal spray in 2015. Narcan^®^ became the most prescribed naloxone product by 2016 [7], although access to and market saturation of overdose reversal products continue to be insufficient, particularly for marginalized persons [8]. In 2021, an 8-mg nasal spray was approved by the FDA (Kloxxado^®^) followed by an injectable, 5-mg formulation (Zimhi™).

These higher-dose products are being developed in part to address high-potency fentanyl and fentanyl analogues in the illicit drug supply [9]. Consensus on the need for higher-dose formulations, however, is lacking due to an array of pharmacokinetic factors (e.g., narrow response window due to rapidity of fentanyl-mediated overdose; adulterants that are non-responsive to opioid antagonists) and behavioral response factors (e.g., subjective dosage protocols and fidelity to dosing protocols) [10, 11]. High systemic levels of synthetic opioids may warrant higher naloxone doses, and higher-dose products may be more beneficial by preventing the need for repeated administration of lower-dose products, especially when quick response and reversal is critical to an individual’s survival. However, a primary concern with using high-dose naloxone for opioid overdose reversal is severe precipitated withdrawal in individuals who are opioid-dependent. Experiencing withdrawal is associated with opioid use to treat symptoms, thereby placing individuals at subsequent risk of overdose [12, 13]. Caution has been raised that precipitated withdrawal induced by higher-dose naloxone products may negatively impact naloxone acceptance among people who have experienced or may experience an overdose reversal [14].

Product features such as dose and route of administration might also influence bystanders’ willingness to carry and administer naloxone. Bystanders encompass a broad range of persons including individuals who use drugs, family, friends, and passersby. For example, bystanders might be concerned that a lower dose may be less effective if fentanyl is involved yet might worry that a higher dose poses an increased risk of adverse withdrawal effects. Similarly, while injectable medications are commonly administered by emergency medical services and healthcare personnel, layperson bystanders may not have experience administering injections, may have trypanophobia (i.e., fear of needles) and/or concern over a potential needlestick and blood-borne pathogen exposure [15]. To ensure bystanders’ willingness to carry and administer naloxone in response to a suspected overdose, it is critical to select products for community distribution with the highest likelihood of being utilized.

State agencies and harm reduction organizations purchasing similarly priced naloxone for community OEND programs are faced with a challenge when deciding which naloxone products to purchase and provide, as the education and training information needed varies depending on the formulation distributed. Additionally, for community OEND programs to succeed in reducing overdose mortality, bystanders, in addition to people who use drugs, must understand how to use the product and be willing to administer it in the setting of a suspected overdose. The aims of the present study were to (1) assess bystanders’ preference for and willingness to administer three FDA-approved naloxone products and (2) identify product features driving preference. This field experiment was conducted as part of a large community OEND event held by a state agency to support statewide naloxone distribution program.

## Methods

The Kentucky Opioid Response Effort (KORE), funded by the Substance Abuse and Mental Health Services Agency (SAMHSA) through the State Opioid Response Grant, has an established program to support OEND efforts statewide. As part of those efforts, KORE staff in partnership with the Kentucky Department for Public Health provided OEND at the Kentucky State Fair, offering units of all three FDA-approved naloxone products at no cost to individuals who receive bystander training either by interacting with a trainer or by reviewing a training video.

### Video development

The KORE opioid overdose response training video was produced by a pharmacist and pharmacy students at the University of Kentucky (UK) and was reviewed and approved by harm reduction experts at KORE and UK. The plain-language video reviews information about opioid safety, opioid overdose response with naloxone, and administration of FDA-approved nasal and injectable naloxone devices. The training video is 8 min long and includes English narration, text slides, and demonstrations of overdose response and proper naloxone administration. To facilitate product flexibility, the video is modular, with general information presented first, followed by standardized dosage-form-specific and product-specific modules that can be utilized in whole or in part depending on the products available and study staff selection. The training video is provided in the Supplement.

### Participant recruitment

For the present study, individuals attending the Kentucky State Fair, held August 18–28, 2022, in Louisville, Kentucky, were invited to receive OEND training and participate in this survey study. Individuals approaching the OEND booth located in the health section of the exposition center were asked if they would be willing to participate in a survey study following completion of the overdose education training; those responding affirmatively were confirmed by self-report to be 18 years or older. Individuals agreeing to participate in the survey study were required to watch the KORE training video, including the modules for all three FDA-approved products, and could not opt for verbal training. Participants were provided with disposable headphones and the video was played on an iPad. To minimize order effects and recency bias, iPads were set up to present the three product-specific modules in different orders. The survey was presented immediately following completion of the training video; all questions were voluntary, no personally identifiable information was collected, and no incentives were provided for completing the survey. We aimed to recruit 600 participants over 10 days at the Fair to ensure we received 400 completed surveys; power curves indicated that 370 participants would provide 80% power to detect significant preference above indifference assuming a product preference of 40%.

The study was deemed exempt by both the University of Kentucky and Kentucky Cabinet for Health and Family Services Institutional Review Boards.

### Survey instrument

Study data were collected and managed using REDCap electronic data capture tools hosted at the University of Kentucky [16, 17]. In addition to demographic information (county of residence, age range, race/ethnicity, and gender), the survey asked participants whether they had ever been trained in opioid overdose recognition and bystander response, currently or had ever carried naloxone, and ever administered naloxone to reverse an overdose. Race and ethnicity were collected via participant self-report to characterize the study sample (see Table 1 for race classification options). Next, participants were provided with the following hypothetical scenario and asked to rate their willingness to administer (0-not at all willing to administer to 10-very willing to administer) each naloxone product.

Assume you are in a public place (like the grocery store), and you come upon a stranger that you believe may have overdosed. Now that you have received bystander training and learned about the individual naloxone products, how willing are you to administer each of them to reverse an opioid overdose?

**Table 1:** Demographics and naloxone experience by product choice

	Total	Higher-dose nasal spray	Lower-dose nasal spray	Intramuscular injection	*p*-value^a^
	(*N* = 509)	(*N* = 226)	(*N* = 130)	(*N* = 153)	
How old are you?					0.304
18–24	81 (15.9%)	40 (17.7%)	19 (14.6%)	22 (14.4%)	
25–34	107 (21.0%)	50 (22.1%)	24 (18.5%)	33 (21.6%)	
35–44	82 (16.1%)	28 (12.4%)	21 (16.2%)	33 (21.6%)	
45–54	78 (15.3%)	42 (18.6%)	19 (14.6%)	17 (11.1%)	
55–64	100 (19.6%)	44 (19.5%)	30 (23.1%)	26 (17.0%)	
65 or older	57 (11.2%)	22 (9.7%)	16 (12.3%)	19 (12.4%)	
Prefer not to answer	1 (0.2%)	0 (0%)	0 (0%)	1 (0.7%)	
Missing	3 (0.6%)	0 (0%)	1 (0.8%)	2 (1.3%)	
To which gender identity do you most identify?					0.405
Female	339 (66.6%)	148 (65.5%)	86 (66.2%)	105 (68.6%)	
Male	152 (29.9%)	66 (29.2%)	40 (30.8%)	46 (30.1%)	
Transgender female	0 (0%)	0 (0%)	0 (0%)	0 (0%)	
Transgender male	4 (0.8%)	3 (1.3%)	0 (0%)	1 (0.7%)	
Gender variant/non-conforming	10 (2.0%)	6 (2.7%)	4 (3.1%)	0 (0%)	
Another option not listed	0 (0%)	0 (0%)	0 (0%)	0 (0%)	
Prefer not to answer	2 (0.4%)	1 (0.4%)	0 (0%)	1 (0.7%)	
Missing	2 (0.4%)	2 (0.9%)	0 (0%)	0 (0%)	
What is your race/ethnicity? (select all that apply)					
American Indian or Alaska Native	13 (2.6%)	6 (2.7%)	4 (3.1%)	3 (2.0%)	0.881^b^
Asian	8 (1.6%)	5 (2.2%)	3 (2.3%)	0 (0%)	0.139^b^
Black or African-American	32 (6.3%)	13 (5.8%)	9 (6.9%)	10 (6.5%)	0.898^b^
Hispanic or Latino	17 (3.3%)	6 (2.7%)	5 (3.8%)	6 (3.9%)	0.700^b^
Native Hawaiian or other Pacific Islander	1 (0.2%)	0 (0%)	0 (0%)	1 (0.7%)	0.556^b^
White	436 (85.7%)	198 (87.6%)	105 (80.8%)	133 (86.9%)	0.180^b^
Prefer not to answer	11 (2.2%)	4 (1.8%)	2 (1.5%)	5 (3.3%)	0.583^b^
Other than the training you took today, have you ever been trained in bystander recognition and opioid overdose response?					0.893
Yes	191 (37.5%)	86 (38.1%)	47 (36.2%)	58 (37.9%)	
No	309 (60.7%)	134 (59.3%)	82 (63.1%)	93 (60.8%)	
I'm not sure	8 (1.6%)	5 (2.2%)	1 (0.8%)	2 (1.3%)	
Missing	1 (0.2%)	1 (0.4%)	0 (0%)	0 (0%)	
Have you ever administered naloxone to reverse an overdose?					0.787
Yes	63 (12.4%)	27 (11.9%)	15 (11.5%)	21 (13.7%)	
No	439 (86.2%)	195 (86.3%)	115 (88.5%)	129 (84.3%)	
I'm not sure	2 (0.4%)	2 (0.9%)	0 (0%)	0 (0%)	
Missing	5 (1.0%)	2 (0.9%)	0 (0%)	3 (2.0%)	
Do you carry naloxone for overdose reversal now, or have you carried naloxone before?					0.336
I have never carried naloxone and do not now	366 (71.9%)	158 (69.9%)	98 (75.4%)	110 (71.9%)	
I have carried naloxone before but do not now	86 (16.9%)	42 (18.6%)	21 (16.2%)	23 (15.0%)	
I carry naloxone now	46 (9.0%)	21 (9.3%)	7 (5.4%)	18 (11.8%)	
I'm not sure	8 (1.6%)	3 (1.3%)	4 (3.1%)	1 (0.7%)	
Missing	3 (0.6%)	2 (0.9%)	0 (0%)	1 (0.7%)	

Demographics and naloxone experience as a function of product choice (*n* = 509)

^a^*p*-value obtained from chi-square/Fisher’s exact tests, as appropriate

^b^Comparison between selected and unselected for each race/ethnicity among three groups

Naloxone products were randomized based on the second-level time stamp the individual started the survey. Participants were then asked which product they would like to take home with them, with product names and equally sized product pictures presented in random order. Finally, participants rated how each of the following factors contributed to their decision using a 0–10 sliding scale (0—did not influence my choice at all to 10—greatly influenced my choice):

- Dose of naloxone in the product
- Ease of use
- Familiarity with the product (i.e., “I have heard of this product before”)
- Fear of needles
- Concern for injuring myself with the used needle
- Potential side effects

Following completion of the survey, participants selected one of the three products to take home and actual product choice was recorded in REDCap by study personnel.

### Statistical analysis

Participant characteristics and responses were summarized and compared among three groups of participants based on the naloxone products they preferred to take home. Categorical variables were reported using frequencies and column percentages. Normally distributed continuous variables were reported using means and standard deviations (SD). Otherwise, medians and first/third quartiles [*Q*1,* Q*3] were reported. The results of the three groups were compared using chi-square/Fisher’s exact tests (categorical variables), ANOVA (normally distributed continuous variables), or Kruskal–Wallis tests (non-normally distributed continuous variables), as appropriate. For each response regarding naloxone experience or factors influencing choice, if the overall test was significant, a subsequent pairwise comparison between three groups was performed with Bonferroni correction.

The statistical significance level was set at *p* < 0.05. To examine potential impact of the order effect on product choice, a chi-square test was used to compare the distribution of product choices among six groups with different presentation orders.

All analyses were conducted in *R* (R Core Team 2022, Vienna, Austria) statistical software.

## Results

The sample included 509 recruited participants. Of these, 226 (44.4%) participants preferred taking home the higher-dose nasal spray, 153 (30.1%) preferred the intramuscular injection, and 130 (25.5%) preferred the lower-dose nasal spray. There were no order effects observed in the product chosen (*p* > 0.05). Concurrence in choice between the product selected in the survey and actual product choice was generally high (higher-dose nasal spray = 92.5%; lower-dose nasal spray = 83.1%; intramuscular injection = 96.1%). Willingness to administer the product to reverse an opioid overdose was high among all three options: higher-dose nasal spray (10.0 [8.0, 10.0]), lower-dose nasal spray (10.0 [8.0, 10.0]), and intramuscular injection (9.0 [5.0, 10.0]).

Demographics and naloxone experience are reported in Table 1. Most participants were age 25–34 (21.0%) or 55–64 (19.6%), with 66.6% being females and the majority being white (85.7%). Similar age, gender, and race distributions were seen among those who chose each product (Table 1). At the time of training, 37.5% reported previous training in opioid overdose response; however, only 9.0% carried naloxone at the time of the study. One in eight (12.4%) reported having previously administered naloxone. Previous bystander training in opioid overdose response did not differ significantly as a function of product preference (*p* = 0.893; Table 1).

Factors most influencing participant product choice were ease of use (median [*Q*1,* Q*3]: 9.0 [7.0, 10.0]), naloxone dose, (8.0 [5.0, 10.0]), and product familiarity (5.0 [5.0, 9.0]) (Table 2). All factors influencing choice differed significantly between products except for ease of use (*p* = 0.855).

**Table 2:** Product choice by factors influencing choice and willingness to administer

	Total	Higher-dose nasal spray (HS)	Lower-dose nasal spray (LS)	Intramuscular injection (II)	HS versus LS^a^ (*p*-value)	HS versus II^b^ (*p*-value)	LS versus II^c^ (*p*-value)
	(*N* = 509)	(*N* = 226)	(*N* = 130)	(*N* = 153)			
Please rate how each of the following factors influenced your choice to take [self_choice] home with you today							
Dose of naloxone in the product					< 0.001	< 0.001	1
Median [*Q*1,* Q*3]	8.00 [5.00, 10.0]	9.00 [6.00, 10.0]	6.00 [5.00, 9.00]	7.00 [5.00, 9.00]			
Missing	45 (8.8%)	16 (7.1%)	13 (10.0%)	16 (10.5%)			
Ease of use					–^d^	–	–
Median [*Q*1, *Q*3]	9.00 [7.00, 10.0]	9.00 [7.00, 10.0]	9.00 [7.00, 10.0]	9.00 [6.00, 10.0]			
Missing	34 (6.7%)	18 (8.0%)	7 (5.4%)	9 (5.9%)			
Familiarity with the product (i.e., I have heard of this product before)					< 0.001	0.092	< 0.001
Median [*Q*1, *Q*3]	5.00 [5.00, 9.00]	5.00 [2.00, 7.25]	9.00 [5.00, 10.0]	5.00 [5.00, 9.00]			
Missing	52 (10.2%)	26 (11.5%)	9 (6.9%)	17 (11.1%)			
Fear of needles					0.063	0.001	< 0.001
Median [*Q*1, *Q*3]	2.00 [0, 6.00]	3.00 [0, 6.00]	5.00 [0, 9.00]	0 [0, 4.25]			
Missing	43 (8.4%)	20 (8.8%)	10 (7.7%)	13 (8.5%)			
Concern for injuring myself with the used needle					0.201	0.033	< 0.001
Median [*Q*1, *Q*3]	2.00 [0, 5.00]	3.00 [0, 5.00]	5.00 [0, 9.00]	1.00 [0, 5.00]			
Missing	47 (9.2%)	22 (9.7%)	13 (10.0%)	12 (7.8%)			
Potential side effects					0.002	1	0.038
Median [*Q*1, *Q*3]	3.00 [0, 5.00]	2.00 [0, 5.00]	5.00 [0.250, 5.75]	2.00 [0, 5.00]			
Missing	64 (12.6%)	29 (12.8%)	16 (12.3%)	19 (12.4%)			
How willing are you to administer each of them to reverse an opioid overdose?							
Higher-Dose Nasal Spray					0.125	0.03	1
Median [*Q*1, *Q*3]	10.0 [8.00, 10.0]	10.0 [9.00, 10.0]	10.0 [8.00, 10.0]	10.0 [7.00, 10.0]			
Missing	39 (7.7%)	15 (6.6%)	12 (9.2%)	12 (7.8%)			
Lower-Dose Nasal Spray					–	–	–
Median [*Q*1, *Q*3]	10.0 [8.00, 10.0]	10.0 [8.00, 10.0]	10.0 [9.00, 10.0]	10.0 [7.50, 10.0]			
Missing	38 (7.5%)	20 (8.8%)	8 (6.2%)	10 (6.5%)			
Intramuscular Injection					1	< 0.001	< 0.001
Median [*Q*1, *Q*3]	9.00 [5.00, 10.0]	8.00 [5.00, 10.0]	8.00 [5.00, 10.0]	10.0 [9.00, 10.0]			
Missing	51 (10.0%)	27 (11.9%)	19 (14.6%)	5 (3.3%)			

^a^The pairwise comparison between higher-dose nasal spray and lower-dose nasal spray (based on the naloxone products preferred taken home)

^b^The pairwise comparison between higher-dose nasal spray and intramuscular injection (based on the naloxone products preferred taken home)

^c^The pairwise comparison between lower-dose nasal spray and intramuscular injection (based on the naloxone products preferred taken home)

^d^The overall test among three groups were insignificant (*p* > 0.05); thus no subsequent pairwise comparison was conducted

The factors influencing naloxone choice differed across the three groups in pairwise comparisons (Table 2). Participants who preferred taking home the higher-dose nasal spray rated the influence of the naloxone dose (9.0 [6.0, 10.0]) significantly higher than those who preferred the lower-dose nasal spray (6.0 [5.0, 9.0]) or the intramuscular injection (6.0 [5.0, 9.0]), respectively, (*p* < 0.001). Familiarity with the product was of significantly higher influence (*p* < 0.001) among participants who chose the lower-dose nasal spray (9.0 [5.0, 10.0]) compared to both those who chose the higher-dose nasal spray (5.0 [2.0, 7.3]) and those who chose the intramuscular injection (5.0 [5.0, 9.0]). Participants who preferred the intramuscular injection rated fear of needles (0.0 [0.0, 4.25]) significantly lower (*p* < 0.001) than those who preferred the higher-dose nasal spray (3.0 [0.0, 6.0]) or lower-dose spray (5.0 [0.0, 9.0]) and concern for injuring themselves with the used needle (1.0 vs. 3.0 [*p* = 0.03] vs. 5.0 [*p* < 0.001]). Participants who preferred the lower-dose nasal spray rated the influence of potential side effects (5.0 [0.25, 5.75]) higher compared to participants who preferred the other two products (2.0 [0.0, 5.0). Though participants’ willingness to administer the three products to reverse an opioid overdose was high, participants who preferred nasal spray were significantly less willing to administer the intramuscular injection compared to those who preferred the intramuscular injection (median: 8.0 vs. 8.0 vs. 10.0).

## Discussion

The opioid overdose epidemic has presented a unique opportunity for bystanders to actively engage in harm reduction strategies by reversing an opioid-related overdose. As such, understanding the factors that facilitate bystander willingness to carry and administer naloxone is critical. Moreover, with the FDA approval of the first over-the-counter opioid reversal medication in 2023, a comprehensive public health strategy must not only consider price and availability of naloxone which are key drivers of choice, but also product features that influence bystander behavior. Although the feasibility of naloxone was studied as part of the FDA approval process for individual formulations, this is first to report a cross-product human factors comparison of bystander naloxone preference. This study contributes to the literature by describing bystander preference to carry and administer various currently approved naloxone products, in the context of community-based distribution, the most common method of naloxone distribution.

Importantly, willingness to administer naloxone was high among all three products, despite their differences. In addition, variability in willingness to administer different products was notably low. The higher-dose nasal spray was selected most often, followed by the intramuscular injection and lower-dose nasal spray with no single product being selected by the majority of participants. This preference variation is promising, as having product choice might encourage bystanders to carry overdose reversal products. In the present study, for example, only 25.9% of the study sample reported currently or previously carrying naloxone. Strategies to increase the number of bystanders willing not just to administer but to carry naloxone is critical to a comprehensive overdose prevention strategy. Interestingly, naloxone experience, either by having previously or currently carrying naloxone, as well as training, was not associated with product choice. Rather, choice differed as a function of product familiarity, fear of needles/concern over needle stick, dose, and potential side effects.

Product or brand familiarity is generally associated with predilection (i.e., familiarity preference) but also interacts with task and context effects to drive decision making [18]. Given the predominance of the name brand Narcan^®^, both in length of time in the market and market share (Freeman et al., 2018), the importance of product familiarity was significantly associated with influencing choice of the lower-dose intranasal product. Notably, “Narcaned” has become a verb in the US-English lexicon to refer to the act of reversing an overdose. In contrast to product familiarity, novelty preference is also a relevant driver of consumer decision making [19]. As a related but distinct factor from product familiarity and novelty preference, the market release date of each product was stated in the study training video. These facts were included because newness-to-market might often come up during a community-based OEND that includes product choice. Although novelty preference and recency to market were not directly assessed, three-quarters of participants selected the two new-to-market products.

Participants choosing the higher-dose nasal product rated higher influence of product dose and lower influence of potential side effects compared to participants selecting other products. Likewise, participants selecting the lower-dose nasal product rated the risk of side effects as more impactful in decision making than those who selected the newer, higher-dose products. Although differential sensitivity to side effects was detected, the low overall rating (3/10, compared to 8/10 for dose) indicates the relatively low influence of side effects in bystander product preference. As the training video was designed to be neutral in its presentation of product side effect risks, study findings may have differed if participants were exposed to additional content discussing risk for adverse reactions (e.g., precipitated withdrawal, pulmonary edema) and the potential implications for subsequent opioid use (see Additional file 1 for training video script).

Almost one-third of participants preferred the intramuscular injection. Participants selecting this product rated its ease of use and dose as most highly influencing their choice; fear of needles and concern over needlestick injuries were rated lowest by these participants. Study staff described interactions following the OEND protocol in which participants disclosed their rationale for selecting the intramuscular injection. In some cases, participants stated that they or a family member already had the lower-dose nasal spray and therefore wanted to take home a different option. Other participants expressed concern over being in close proximity to someone’s face due to risk of infectious disease as well as potential emesis. As this study took place during the time of the COVID-19 declaration of a national emergency, precaution against respiratory transmission of infectious diseases was likely higher than in the pre-pandemic era. Furthermore, some participants likened the use of the intramuscular naloxone injector to their familiarity with epinephrine injection devices.

Although product selection did not differ significantly as a function of age and gender, the study sample provides relevant context to the present findings. First, the relatively even distribution of participation across age ranges from 18 to 65 or older supports the generalizability of study findings, although the higher proportion of female participants is noteworthy. Race distribution generally mapped on to state’s demographic population rates, resulting in an insufficient sample size to test race, ethnicity, and other intersectional differences between groups. Documented disparities in overdose response training among people of color who inject drugs attributed to systematic racism warrant evaluation of bystander training as well [20, 21].

As this was a convenience sample, self-selection to approach the harm reduction unit and participate in OEND likely explains to some degree the relatively high bystander willingness to carry and administer naloxone. However, most individuals in the study sample had never received bystander overdose response training or carried or administered naloxone. Although Kentucky had the second highest rate of fatal overdose in the US in 2020 [22], the frequency of overdose response experience was remarkably high. From a public health perspective, understanding the proportion of bystanders that utilize their overdose response training as well as administer naloxone will fulfill an urgent need to improve the efficiency and effectiveness of bystander OEND programs.

While this study provides insight into the general public’s perception of various naloxone products, it should be replicated in a sample of people who use opioids and other drugs. The extent to which the opinions of this bystander sample align with or diverge from those of people with lived experience (i.e., prior overdose and/or increased likelihood of having witnessed an overdose) is unclear. However, in a survey of 1152 individuals entering opioid use disorder treatment, respondents either had no preference (48.4%) or preferred a higher-dose formulation (35.9%) if personally experiencing an overdose [23]. Qualitative interviews documenting reasons why people who use opioids might not accept or carry naloxone identified the reluctance of individuals to make someone feel the effects of a precipitated withdrawal tempered by the magnitude and time-limited nature of the aversive state [24]. Although not included in the present study of bystanders, future product preference studies conducted among people with lived experience should also include the choice of generic intramuscular naloxone injections, which provides the benefit of titrated dosing.

The hypothetical scenario utilized in the study is both a strength and limitation of the design. A standardized scenario increased the likelihood that each participant generally considered the same context during decision making, although personal experience likely also influenced choice. In addition, selecting a stranger as opposed to a family or friend was intended to decrease the variability in the social distance of the target between participants (e.g., to standardize this social distance). The public context of the scenario might have increased social desirability bias; however, all participants were provided privacy when responding and responses were not immediately known to the present staff. This feature also more closely approximates encountering overdoses in public as a real-world scenario.

## Conclusions

The findings from this study suggest that, in the general population, there is a high willingness to administer various naloxone products. However, if given a product choice, bystanders vary in their product preference. In the absence of comparative effectiveness studies, public health agencies should consider bystander preferences when purchasing similarly priced naloxone products for distribution. As the number and variety of available products continue to increase, continuous evaluation of formulation acceptability, especially among individuals with lived experience who are receiving and administering overdose reversal agents, is critical to support adoption and save lives.

### Supplementary Information

**Additional file 1. Video**. StateFair_Video_ForWeb. Naloxone Training Video. Naloxone training video reviewing information about opioid safety, opioid overdose response with naloxone, and administration of FDA-approved nasal and injectable naloxone devices.

## Data Availability

All data analyzed during this study are included in this published article.

## Abbreviations

KORE: Kentucky Opioid Response Effort
OEND: Overdose Education and Naloxone Distribution
SAMHSA: Substance Abuse and Mental Health Services Agency
FDA: United States Food and Drug Administration
UK: University of Kentucky
